# Radiological and clinical outcomes of cervical disc arthroplasty for the elderly: a comparison with young patients

**DOI:** 10.1186/s12891-019-2509-0

**Published:** 2019-03-18

**Authors:** Jau-Ching Wu, Hsuan-Kan Chang, Wen-Cheng Huang, Tsung-Hsi Tu, Li-Yu Fay, Chao-Hung Kuo, Chih-Chang Chang, Ching-Lan Wu, Huang-Chou Chang, Henrich Cheng

**Affiliations:** 1 Department of Neurosurgery, Neurological Institute, Taipei Veterans General Hospital, Room 525, 17F, #201, Shih-Pai Road, Sec. 2, Beitou District, Taipei, 11217 Taiwan; 2 School of Medicine, National Yang-Ming University, Taipei, Taiwan; 3 Institute of Pharmacology, National Yang-Ming University, Taipei, Taiwan; 4 Department of Biomedical Imaging and Radiological Sciences, National Yang-Ming University, Taipei, Taiwan; 5 Department of Radiology, Taipei Veterans General Hospital, Taipei, Taiwan; 6 Department of Surgery, Fu Jen Catholic University Hospital, New Taipei City, Taiwan

**Keywords:** Range of motion (ROM), Cervical disc arthroplasty (CDA), Heterotopic ossification (HO), Elderly

## Abstract

**Background:**

This study aimed to investigate whether cervical disc arthroplasty (CDA) would be equally effective in elderly patients as in the young. The inclusion criteria of published clinical trials for CDA-enrolled patients covered the ages from 18 to 78 years. However, there was a paucity of data addressing the differences of outcomes between older and the younger patients.

**Methods:**

A series of consecutive patients who underwent one- or two-level CDA were retrospectively reviewed. Patients at the two extreme ends of the age distribution (≥65 and ≤ 40 years) were selected for comparison. Clinical outcome parameters included visual analog scale (VAS) of neck and arm pain, neck disability index (NDI), and Japanese Orthopaedic Association (JOA) scores. Radiographic outcomes included range of motion (ROM) at the indexed level and evaluation of heterotopic ossification (HO) by computed tomography (CT). Complication profiles were also investigated.

**Results:**

There were 24 patients in the elderly group (≥65 years old) and 47 patients in the young group (≤40 years old) with an overall mean follow-up of 28.0 ± 21.97 months. The elderly group had more two-level CDA, and thus the mean operative time was longer (239 vs. 179 min, *p* < 0.05) than the young group. Both groups had similarly significant improvement in clinical outcomes at the final follow-up. All the replaced disc segments remained mobile on post-operative lateral flexion and extension radiographs. However, the elderly group had a slight decrease in mean ROM (− 0.32° ± 3.93°) at the index level after CDA when compared to that of pre-operation. In contrast, the young group had an increase in mean ROM (+ 0.68° ± 3.60°). The complication profiles were not different, though a trend toward dysphagia was noted in the elderly group (*p* = 0.073). The incidence or severity (grading) of HO was similar between the two groups.

**Conclusions:**

During the follow-up of two years, CDA was equally effective for patients over 65 years old and those under 40 years in clinical improvement. Although the elderly group demonstrated a small reduction of mean ROM after CDA, in contrast to the young group which had a small increase, the segmental mobility was well preserved at every indexed level for each group.

## Background

As the baby boomers have aged, the population above 65 years old has substantially increased for the last ten years, along with an increase in the average life span, in major industrialized countries, and will continue to rise more quickly in the next 30 years. It has been estimated that the global population over 80 years old will triple by 2050 [1]. The need of care in spinal degenerative diseases also has grown enormously in the past decade, along with momentous developments in related technology, and thus the need for such services will probably continue to grow for the next two decades because of this increase in the geriatric population and life span. Since elderly patients are usually perceived as at a higher risk for spinal surgery, spine surgeons would be expected to face challenges from this demographic change and should be prepared.

For more than fifty years, anterior cervical discectomy and fusion (ACDF) has been a widely accepted operation for cervical spine degenerative diseases, including disc herniation and spondylosis, that are refractory to medical management. The high rates of success and patients’ satisfaction with ACDF has made the operation a standard of care in common neurosurgical practice. On the other hand, during the latest decade, cervical disc arthroplasty (CDA) has become a viable option for cervical herniated discs and/or spondylosis. For one- or two-level cervical disc diseases, multiple prospective, randomized, and controlled studies by the United States Food and Drug administration-investigational device exemption (US FDA-IDE) have successfully demonstrated the effectiveness and safety of CDA in comparison to ACDF [2–10]. Moreover, these reports have proven that CDA devices can maintain segmental mobility at the indexed level(s) and likely have the potential to reduce adjacent segment disease (ASD) [11].

The published FDA trials of CDA report on enrolled adult patients of a surprisingly wide range of ages, from 18 to 78 years, depending on different trials, although the mean age was approximately mid-forties [3, 12–14]. The outcomes of anterior cervical arthrodesis have been extensively surveyed in aged cohorts and the literature has suggested that advanced age is a predictive factor of complications, including dysphagia, re-admission, and longer length of hospital stay [15]. Despite the growing popularity of CDA, there has been a substantial lack of studies focusing on older vis-à-vis younger patients who underwent the surgery. When it came to the question of age affecting the outcomes of CDA, the answers remained elusive, since no evidence could be extracted from existing randomized and controlled trials [16].

The present study, therefore, aimed to investigate the differences of adopting CDA between elderly and young patients. A retrospective analysis was conducted to compare the clinical and radiological outcomes among CDA patients at the two ends of the age distribution. We hypothesized that the radiological and clinical outcomes would be similar between the elderly and young groups.

## Methods

### Inclusion and exclusion criteria

This was a retrospective study that reviewed medical charts and radiological images in detail for patients’ data and characteristics. Consecutive adult patients (> 18 years of age) who underwent one- or two-level CDA with Prestige LP artificial discs (Medtronic, Memphis, TN) at the subaxial (C3–7) cervical spine in a single institute were included. The surgical indication for CDA was symptomatic cervical disc herniation and/or spondylosis causing radiculopathy, myelopathy, or both, that was refractory to medical treatment. All patients had failed at least 12 weeks of non-operative management, including physical and pain control therapy, but remained medically intractable prior to surgery. Exclusion criteria were: [1] spinal trauma and fracture; [2] evident segmental instability (i.e. more than 3.5 mm translation or 20° angular motion); [3] arthrodesis without mobility; [4] severely incompetent facet joints; [5] adjacent segment disease; [6] ossification of posterior longitudinal ligament (OPLL); [7] kyphotic deformity; [8] infection; and [9] long-term steroid use. Chronic systemic diseases, including severe osteoporosis, malignancy, metabolic bone disease, autoimmune disease or spondyloathropathy such as rheumatoid arthritis or ankylosing spondylitis, cerebrovascular disease, were also excluded from the current study.

Since the study aimed to investigate the age-related effects of CDA surgery, patients at the two ends of the cohort were extracted for comparison. The patients who were aged 65 or more years and those 40 years or less were grouped into two: the elderly group (≧65 years) versus the younger group (≤40 years).

Magnetic resonance images (MRIs) and X-ray images were obtained of every patient for confirmation of the diagnoses. Also, pre-operative computed tomography (CT) scanning for the evaluation of bone spur, calcified disc, or OPLL (for exclusion), was also a routine examination for all patients before the CDA surgery in the current series.

### Surgical technique

The standard Cloward approach for anterior cervical discectomy was executed in all patients [17]. In addition to thorough discectomy, bilateral uncovertebral joints and bone spurs were removed extensively with drilling burrs or Kerrison’s rongeurs to achieve generous decompression of the dura sac and nerve roots. Also, the posterior longitudinal ligaments were always resected to ensure adequate decompression. Upon placement of the Prestige LP artificial disc, meticulous endplate preparation, selection of a proper fitting size, and centering of the device were considered imperative to minimize the chances of heterotopic ossification (HO) formation [18]. Furthermore, we used copious saline irrigation persistently to wash away the bone dust generated from osteophyte drilling in every case. All surgeries were done by three experienced neurosurgeons (JC Wu, WC Huang, and H Cheng) with consistent techniques detailed in our previous publications [19–24].

### Clinical and radiographic follow-up

**Fig. 1:**
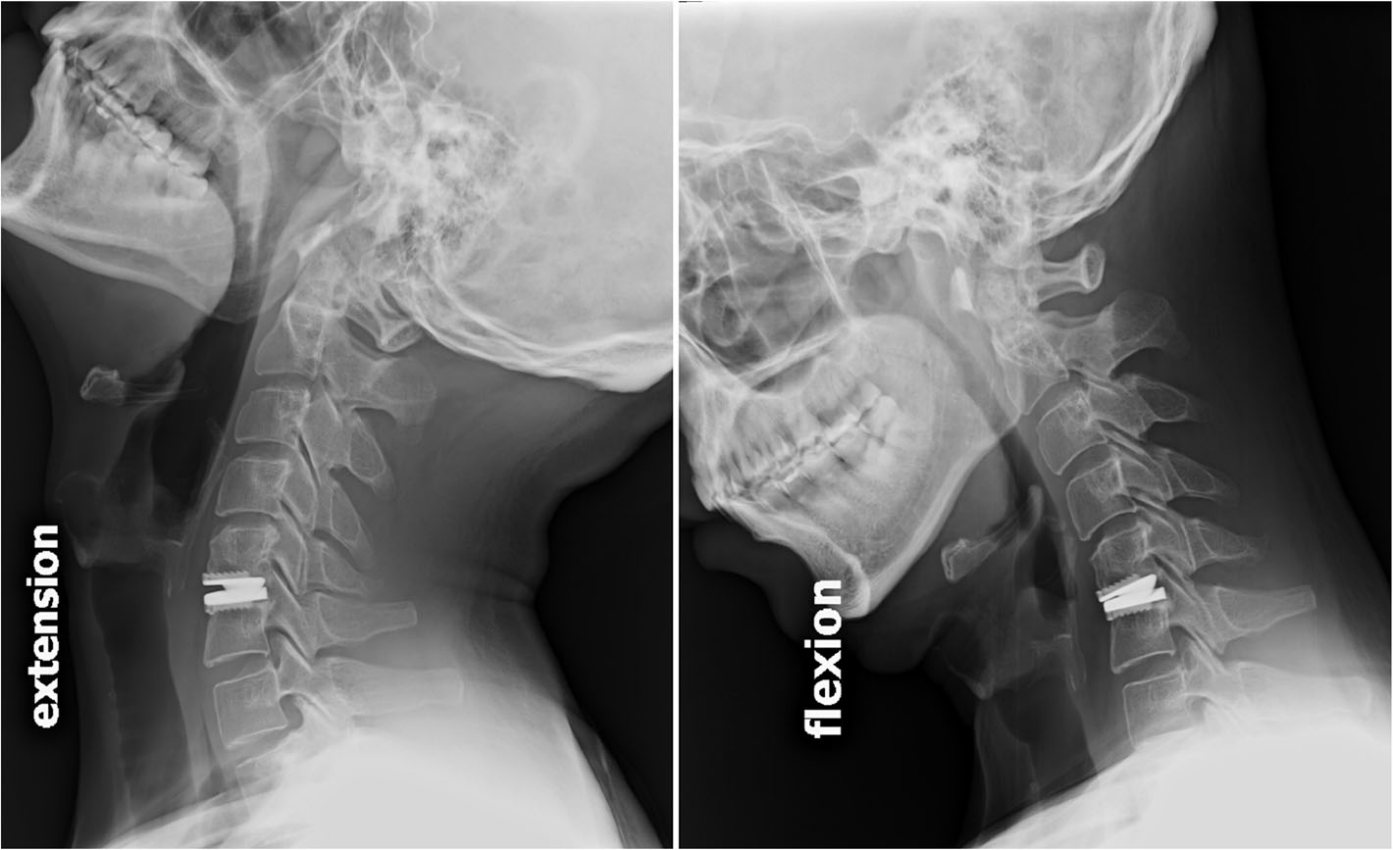
Segmental range of motion (ROM) at the index level on standing lateral flexion/extension X-ray films at post-operative 24-months follow-up from a male patient in the young group (≤40 years old)

Regular visits at the outpatient department were arranged at pre-operation, and post-operative 6-weeks, and at 3, 6, 12 and 24 months for all patients. Clinical follow-up parameters included visual analog scales (VAS), neck disability index (NDI), and modified Japanese Orthopaedic Association (JOA) scores, which were collected by experienced physician assistants during regular post-operative follow-ups. Routine X-ray images including antero-posterior, lateral, and flexion-extension films were taken at every regular visit at the clinic. Radiological criteria for adjacent segment degeneration (ASD) in X-ray films were the presence of disc space narrowing, osteophytes, or sclerosis of the endplates [25]. Incidences of HO formation were accessed by not only the post-operative lateral radiographs but also by CT scans with three-dimensional reconstruction, and graded according to the McAfee’s classification [26]. Segmental range of motion (ROM) at the index level was determined with standing lateral flexion/extension radiographs (Fig. 1) at post-operative 24-months follow-up using the Cobb method [2, 24]. Radiological measurements were completed by a board-certified neuroradiologist independently using the PACS system software, SmartIris (Taiwan Electronic Data Processing Co., Taiwan).

### Statistical analysis

Paired t-tests and independent t-tests were used for continuous variables analysis. Categorical variables were compared via Pearson’s chi-square test. All statistical analysis were conducted using the SPSS Software (SPSS Inc., Chicago, IL). The statistical significance was set at *p* < 0.05.

## Results

### Demographics and patient-reported clinical outcomes

A total of 171 patients underwent 1- or 2- level CDA with Prestige LP artificial discs, with an average age of 48.3 ± 9.99 years at the time of operation. In order to investigate the age-related effects of CDA, the current study aimed to analyze patients at the two ends of the age distribution in the cohort. Therefore, the study included 71 patients who were aged 65 years or more and those less than 40 (to a minimum of 18 years), to minimize the bias from mid-aged CDA patients. The demographic data of these are demonstrated in Table 1.

**Table 1:** Demographic data

	Elderly group (≧65 year-old)	Young group (≤40 year-old)	*P* value
Case number	*n* = 24	*n* = 47	
Age (years)^a^	71.2 ± 4.79 (65–80)	33.9 ± 4.45 (23–39)	< 0.001*
Male:Female	13:11	31:16	
Follow-up (months)^a^	22.6 ± 22.29	30.8 ± 21.52	0.145
Total levels	40	57	
one-level	8	37	
two-level	16	10	
Level distribution			0.253
C3–4	4	2	
C4–5	15	16	
C5–6	17	35	
C6–7	4	4	
Blood loss (ml)^a^	171.4 ± 164.75	115.2 ± 131.41	0.178
Operative time (min)^a^	239.4 ± 81.40	178.6 ± 58.00	0.008*

^a^Values are presented as mean ± SD (standard deviation)

*p < 0.05;

The patients were divided into two groups: the elderly group (≥65 years old) consisted of 24 patients with a mean age of 71.2 ± 4.79 years; the young group (≤40 years) composed of 47 patients with a mean age at 33.9 ± 4.45 year-old. The mean follow-up duration was 28.0 months, without differences between the two groups (averaged 22.6 versus 30.8 months, elderly vs. young, respectively). There were more two-level cases in the elderly group than the young group, probably due to more spondylosis as age advanced. The most commonly indexed levels of CDA were C4–5 and C5–6, accounting for 85% of the entire series. Although there were no differences in terms of level of distribution and blood loss, the elderly group had a longer operative time than the young group of patients (239.4 ± 81.40 versus 178.6 ± 58.00 min, *p* = 0.008*). It was not clear what caused the discrepancy of time, approximately 50 min, consumed for the CDA surgery in the two age groups. The clinical outcomes, including VAS of neck and arm pain, NDI, and JOA scores were all similarly satisfactory after the surgery, during the follow-up. All the patient-reported outcome parameters had significant improvement at the final follow-up, when compared to the pre-operative scores (Table 2).

**Table 2:** Clinical outcomes

	Mean pre-op VAS neck (SD)	Final follow-up (SD)	*P* value	Mean pre-op VAS arm (SD)	Final follow-up (SD)	*P* value
Elderly group	4.9 (2.67)	2.0 (1.70)	0.002*	4.5 (3.37)	1.9 (2.07)	0.013*
Young group	3.1 (2.68)	2.0 (2.12)	0.020*	2.7 (2.70)	1.0 (1.75)	0.004*
	Mean pre-op NDI (SD)	Final follow-up (SD)	*P* value	Mean pre-op JOA (SD)	Final follow-up (SD)	*P* value
Elderly group	19.2 (12.44)	8.6 (6.50)	0.012*	10.0 (4.45)	13.14 (2.79)	0.009*
Young group	9.9 (7.90)	6.0 (4.34)	0.014*	13.3 (1.67)	16.0 (1.19)	<0.001*

*VAS* Visual Analog scale for pain, *NDI* Neck Disability Index, *JOA* Modified Japanese Orthopaedic Association score, *SD* standard deviation

P value: pair-t test compared to pre-operative scores. *p < 0.05

### Complications

The complication profile is displayed in Table 3. There were 3 cases of dysphagia, 1 case of dysphonia, 1 case of C5 palsy in the elderly group (*n* = 24), and 1 case of dysphagia, 1 case of dysphonia, and no cases of C5 palsy in the young group (*n* = 47). There was a slightly higher incidence of dysphagia in the elderly group, but it reached no statistical significance when compared to the young group (*p* = 0.073). All the patients experienced temporary deficit of various lengths (weeks to months) but all resolved spontaneously without permanent neurological dysfunction within 6 months (the latest recovery time). The incidence of radiographic and symptomatic ASD were not different between the two groups. There was no secondary surgery, no implant removal or revision (conversion to ACDF), no neural injury, no wound infection or dehiscence, and no post-operative hematoma in the series during the follow-up period. Furthermore, using the McAfee’s classification, [26] the HO formation was accessed and graded in the current series with post-operative CT scans. Under such scrutiny, approximately 40% of the patients in the series developed HO. Interestingly, although the two groups developed a similar grading of HO, there were more high-grade HOs in the elderly group.

**Table 3:** Complication profile

	Elderly group (*n* = 24)	Young group (*n* = 47)	P value
Dysphagia	3 (12.5%)	1 (2.1%)	0.073
Dysphonia	1 (4.2%)	1 (2.1%)	0.623
C5 palsy	1 (4.2%)	0 (0%)	0.159
ASD			
Radiographic	2 (8.3%)	3 (6.5%)	0.780
Symptomatic	1 (4.2%)	2 (4.2%)	0.972
HO			0.710
Grade 0	21 (58.3%)	25 (54.3%)	
Grade 1	6 (16.6%)	12 (26.0%)	
Grade 2	5 (13.8%)	6 (13.0%)	
Grade 3	4 (11.1%)	3 (6.5%)	

*ASD* adjacent segment degeneration, *HO* Heterotopic Ossification

### Segmental range of motion (ROM)

**Fig. 2:**
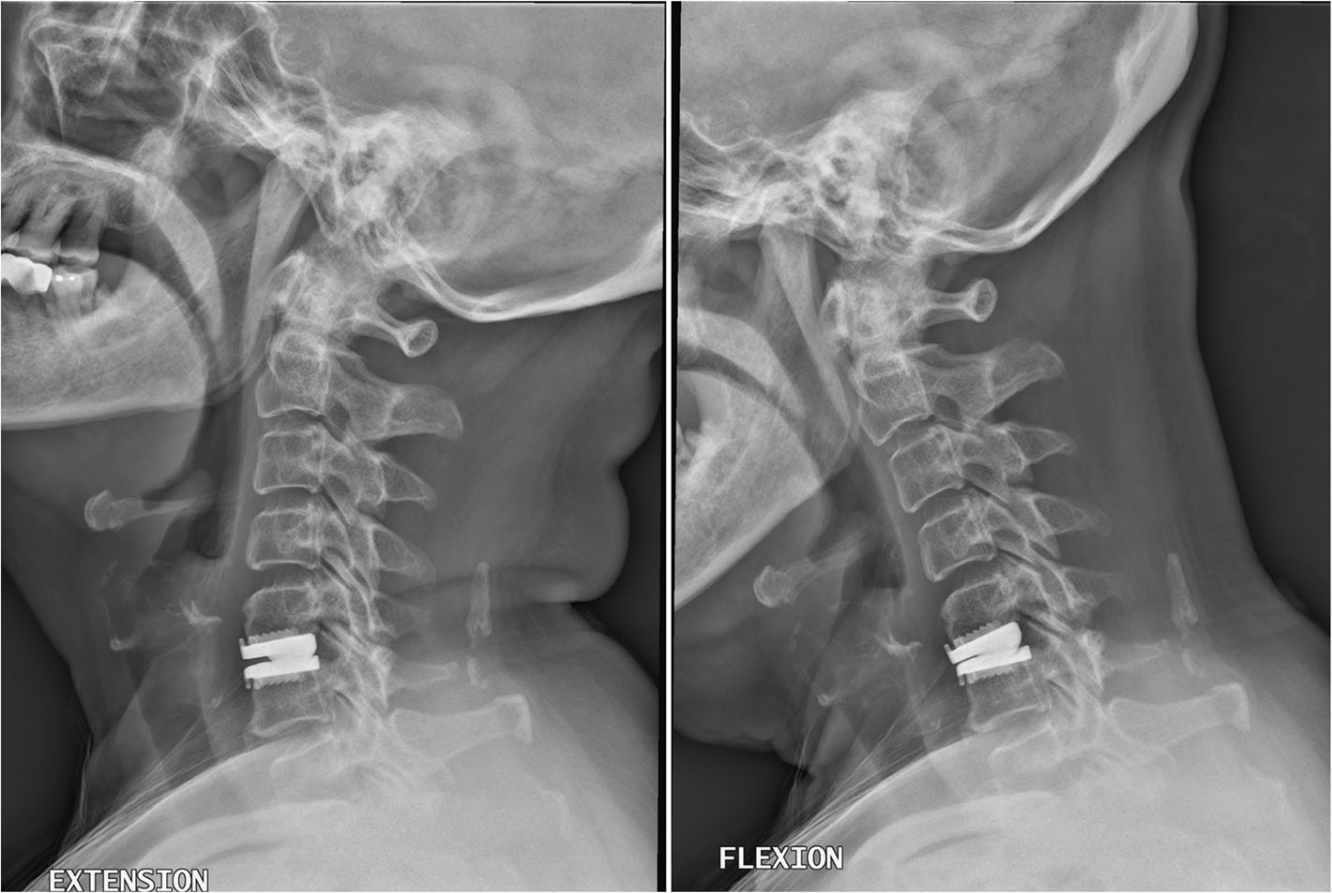
A female patient in the elderly group (≥65 years old), who underwent C5–6 cervical disc arthroplasty (CDA) with Prestige LP disc. The range of motion (ROM) at the index level was well-preserved 2 years after CDA

Young patients tended to have increased segmental ROM after CDA, while the elderly patients had a decrease in segmental ROM after the CDA surgery. Overall, the segmental ROM was well-preserved in all the patients after CDA (Table 4, Fig. 2). However, there was a distinct difference in regard to the change of ROM between the two groups. In the elderly group, the segmental ROM at the indexed level tended to decrease within 24 months after surgery, from 6.1 ± 3.67 degrees pre-operatively to 5.6 ± 3.49 degrees post-operatively. In contrast, for the young group, the segmental ROM increased from 7.8 ± 4.16 to 8.3 ± 4.17 degrees after CDA. In other words, the alteration of segmental ROM after CDA was the major distinction between the elderly patients and the relatively younger ones (decrease vs. increase, elderly versus young).

**Table 4:** Pre- and post-operative ROM in the young and elderly groups

	Pre-operative ROM (degree)^a^	Post-operative ROM (degree)^a^	ΔROM^a^	*P* value^*α*^
Elderly group	6.1 ± 3.67	5.6 ± 3.49	−0.32 ± 3.93	0.464
Young group	7.8 ± 4.16	8.3 ± 4.17	0.68 ± 3.60	0.308
P value^β^	0.036*	0.001*	0.202	

*ROM* range of motion; *p < 0.05

*ΔROM* The difference between pre- and post-operative ROM

^a^Values are presented as mean ± SD

*α* Comparison between pre- and post-operative ROM

*β* Comparison between Elderly and Young group

## Discussion

As medicine, economies and technologies have continued to advance, aging of the population also has accelerated tremendously in the past decades, and there has been a marked reduction of fertility and an extension of life expectancy world-wide [27]. As a consequence, the elderly population (older than 60 years old) has boomed from approximately 200 million in the world 60 years ago to approximately 600 million by the beginning of the twenty-first century. The growth of an aging population has been exponential in recent past years, and this rate of acceleration was estimated to be 3.5 times faster than that of the entire population [28]. One of the major issues of aging has been the remarkable burden of degenerative spinal disorders among the elderly, causing not only back problems but also cervical spondylosis. For example, in one national database, the utilization of anterior cervical procedures had a 28-fold increase for persons 65 years and older during a 15-year period at the end of the last century [29]. The increasing need of surgical care in cervical spine degenerative disease is real and imminent. However, there has been a paucity of literature that has focused on the outcomes of cervical spinal surgery among the geriatric population.

The current study focused on a comparison of patients at both ends of the age distribution (≧65 or ≤ 40 years) in a cohort of CDA patients. Patients who had been followed-up for more than 24 months were included for retrospective analysis of their clinical outcomes (i.e. VAS, NDI, and JOA scores), radiological parameters (i.e. pre-, post-operative ROM, and incidences of HO), and the complication profiles (e.g. dysphagia, dysphonia, and C5 palsy). The study demonstrated small but distinct discrepancies between the elderly and young in the changes of segmental mobility after CDA. More than 2 years after 1- or 2-level surgery for CDA, both groups of patients, regardless of their age differences (i.e. elderly versus young), demonstrated improvements in clinical outcomes when compared to that of pre-operation. Moreover, both groups had successfully preserved segmental mobility at the indexed levels with CDA.

However, in the current study, the most significant differences between the elderly and the young was their segmental mobility. Prior to the CDA surgery, the elderly patients had slightly less ROM than the young. After the CDA, the discrepancy had been magnified even more. The young group had increased ROM compared to that of pre-operation, while, in contrast, the elderly group had a slightly decreased ROM. Although the ROM was still mobile at an average of 5.6° for the elderly, the young group of patients were more mobile with an average ROM of 8.3°.

Also, the incidences and severity of the HO in both age groups were not significantly different. The complication profiles were also very similar among the elderly and young patients.

This paper demonstrated that the application of CDA in the elderly had similar clinical outcomes to the young patients, and thus implied that CDA could be a viable and effective option for patients greater than 65 years old. For the young patients (≤40 years) who needed surgical management for 1- or 2-level cervical disc herniation or spondylosis, CDA would very likely achieve good outcomes and preserve segmental mobility that would be even greater than the elderly group.

There have been many FDA-IDE trials of CDA on 1- and 2-level cervical disc herniation or spondylosis that caused radiculopathy, myelopathy, or both. The trials unanimously demonstrated that CDA was as safe and effective as ACDF in the improvement of neurological symptoms, while superior in preservation of the segmental mobility than ACDF. These trials were commonly designed to enroll adult patients 18 to 78 years old and had mean ages of enrolled subjects around the mid-forties (averaged approximately at 45 years for all studies) [4–6, 13, 30]. However, none of them had investigated the detailed age-related differences, and it remained elusive as to the various effects of CDA among the elderly patients than the younger patients. Although there had not been reports against the use of CDA in patients over 65 years, it has been unclear if these elderly patients do as well as those patients under 40 years of age. Probably the most common concerns against using CDA among the elderly were the risks of complications and the lack of economic efficiency because the elderly patients have a shorter life expectancy than the younger patients. Nevertheless, several FDA-IDE trials had pushed the border of the surgery to use CDA in patients older than 70 years [2, 3, 8, 31].

Ideal candidates of CDA are young patients who have soft disc herniations and little degeneration in the facet joints, and it is commonly accepted that young patients benefit more from CDA because it provides them with more years of neck mobility than ACDF. Reports on CDA in the literature have had various inclusion criteria for age. For instance, the RCTs for Kineflex-C (SpinalMotion, Inc), ProDisc-C (Depuy-Synthes, Inc), and PCM (Nuvasive, Inc) trials set the limitation of age for inclusion to be < 60 or < 65 years old [4, 5, 10]. The Mobic-C (Zimmer-Biomet, Inc) trials allowed patients equal to or less than 69 years old [7, 30]. The clinical trials for Prestige and Bryan (Medtronic, Inc) included patients no older than 78 years [2, 3, 31]. Despite the wide range of age inclusion in these large-scale prospective trials, the mean age of patients enrolled was around 43 to 46 years. In the current study, the two groups had similar demographics, except the distinct differences in their mean ages [71.2 (65-80) versus 33.9 (23-39) years, elderly versus young, respectively]. The study provided a direct comparison of patients of different ages undergoing CDA surgery, and attempted to differentiate the age factor, filling the information gap in the randomized control trials of the FDA mentioned above.

Older age could be associated with cardiopulmonary complications in major surgery but not necessarily with anterior cervical spine surgery. There was one study which utilized the American College of Surgeons’ National Surgical Quality Improvement Program (ACS-NSQIP) database to analyze the complications after ACDF. It demonstrated that, by multivariate logistic regression, aging was associated with pulmonary complications, UTI, cardiac complication, and sepsis [32]. There was also another large cohort study that revealed similar findings for older patients undergoing ACDF [33]. However, in the current study and the FDA trials, there were few cardio-pulmonary or infectious complications. Perhaps the most common complications of anterior cervical spine are dysphagia, dysphonia, and temporary C5 palsy. Likewise in the series, dysphagia accounted for the most common complication at approximately 12 and 2% in the elderly and young groups, respectively. Dysphonia accounted for 4.2 and 2.1% in each of the two groups, respectively. These incidences were comparable with the mean incidences of dysphagia and dysphonia combined from the FDA trials, which ranged from 4.73 to 17.33% [34]. Due to the lack of operational definition and severity of dysphagia, the prevalence varied among different studies. It was reported that patients of old-age (> 70) had a 4.96-times higher risk of severe dysphagia after ACDF in a nationwide-scaled database [35]. There were few data available in the literature for CDA, especially for the elderly recipients. The current report also merits the addressing of these rates of complications after CDA in the elderly. There were only temporary dysphagia and dysphonia in the elderly patients in the series and they were all resolved spontaneously within 6 months.

It might be intuitive to expect higher complication rates for elderly than younger patients who undergo surgery, but this was not the case in anterior cervical discectomy. In the current series of CDA, the complication rates were very similar for the elderly and young patients. There was one patient (2.1%) in the elderly group who developed unilateral C5 palsy after CDA, while there was no C5 palsy in the young group. In the FDA trials, neurologically related adverse events were reportedly ranging from 0.4 to 13.4% in different trials in gross, but the details were not disclosed in the paper [34]. For anterior cervical fusion, the risks of C5 palsy varied from 3 to 17%, depending on the levels of discectomy, corpectomy or not, instrumentation, etc [36]. The incidence of C5 palsy specifically after CDA surgery has never been reported or compared to that after ACDF in the trials. In the current series, the incidences of adverse events in the elderly patients who underwent CDA were similar to that of the young.

The middle-age patients, who were not included in the present study, had very similar outcomes to the elderly and young groups. Those middle-age patients reportedly had common results. For example, one of our previous studies evaluated 50 middle-age patients (mean age 45.6 ± 9.33 years) of 1-level Prestige CDA and demonstrated similar clinical improvements at 24 months after surgery. All the mean VAS, NDI, and JOA scores of the middle age patients improved significantly from 4.4 to 1.8, 15.5 to 8.6, and 13.1 to 14.7, respectively [24]. Furthermore, these results were consistent with that demonstrated in the current study, including all patient-reported outcome parameters (VAS, NDI, and JOA scores) of the elderly and young patients, and unanimously had similar improvements at the final follow-up. Many of our published studies have focused on the most commonly reported middle age groups (with approximately the same mean ages around 45 to 50 years), indicating insignificance from the two extreme age groups, the elderly and young [18–20, 22, 37–41].

There are limitations to the current study. The patients were extracted from a cohort of CDA patients treated surgically by the 3 senior authors for analysis. The elderly patients inevitably had a shorter life expectancy than the young and this could be a confounding factor. The length of follow-up can be a weakness for this study. There was a discrepancy in length of follow-up for individual patients. However, average length of follow-up still achieved 22.6 and 30.8 months in the elderly and young group, respectively. The sample size of both groups was small and lacked a control group of ACDF for comparison. Since the age-related discrepancies appeared to be subtle, a larger sample size could be necessary to detect the subtle differences. Furthermore, the criteria of age used for grouping (≥65 and ≤ 40 years old) in the study was arbitrary and might not be the best way for division. However, since we intended to focus on both ends of the age distribution, especially for the performance of CDA in the elderly patients, middle-aged cases (41–64 year-old) were not in the scope of the present study. Besides, the effectiveness of CDA for middle-aged patients has already been well demonstrated in many Class-I FDA trials. Although the demographic data, other than age, of the two groups showed little differences, the less segmental mobility demonstrated in the elderly was possibly related to degeneration and aging. More adjustment of statistics is usually required to reduce the bias and confounders. Also, the slowly on-going problems of aging and degeneration might require decades before they cause symptoms. Those benefits of CDA utilization in the elderly patients could be masked because the young group had less degeneration and thus required longer follow-up. In contrast, there could be some competing bias in the elderly group that might have skewed the data.

## Conclusion

During the follow-up of 2 years, CDA was equally effective for patients over 65 years old and those under 40 years in clinical improvement. Although the elderly group demonstrated a small reduction of mean ROM after CDA in contrast to the young group, which experienced a small increase, the segmental mobility was well preserved at every indexed level for each group.

## Abbreviations

ACDF: Anterior cervical discectomy and fusion
ACS-NSQIP: American College of Surgeons’ National Surgical Quality Improvement Program
ASD: Adjacent segment degeneration
CDA: Cervical disc arthroplasty
CT: Computed tomography
HO: Heterotopic ossification
JOA: Japanese Orthopaedic Association
MRI: Magnetic resonance image
NDI: Neck disability index
OPLL: Ossification of posterior longitudinal ligament
ROM: Range of motion
US FDA-IDE: United States Food and Drug administration-investigational device exemption
VAS: Visual analog scale
